# Supplementary material for: Chromothripsis during telomere crisis is independent of NHEJ, and consistent with a replicative origin
Source: Genome Res. 2019 May;29(5):737–49. doi: 10.1101/gr.240705.118 (PMC6499312; doi:10.1101/gr.240705.118)
Supplement: Supplemental Material [file supp_gr.240705.118_Supplemental_file_1.zip › contigs/annotated_contigs/DB55/contig.2.DB55_length_751_mean_cov_6.52996005326.docx]

**DB55_length_751_mean_cov_6.52996005326**

```
ACCTTAATGTAACTGGTAGGAAAGA|AGGTTTGAAGGTATACAACCTTTTGAAATTATGTTTCATGAATTTCAAAGAGAAAAAAACAGA
                          >chr7:152032916-152033316 + E=5e-212
TTCAGACTATAGGGTTTTTTGGTTTTTTTTTGTTTTTTAAGAAATAGGATCGGCACCAGATCTGATCCCATAAAGCAGGTAAGAAGACA

GGCTTTCAGAGTTCATCCTGTTCTCTTTTCTCCTCTAGCTATTATTATTACTGGCTCTGCCCATGATTCTAACTTGGATCTGCTAAAGA

AACAGTAGGGAGCGATAGTTTTGGTTTTACTTTTGGTTTAGATTTTGTTGTTTCAGTCTTGTGTTCTTTAACAAATAAAGAGGACTCTA

TGACTGCTTCCTAGATAGCAGTAATTCCTGCCACAAAATAGGAACAAACAACAAAAAAGGTATCACT|AA|CACTCTCACAGACACACT
                                                                    >chr7:152656719-152
CAGGATCAATACTTTGTATCCTTCAATCCAATCCAGTTGACACTCAATATTAACCATCACAGCATGGAAAGACCCTCTTTGCCGTGTGA
657048 + E=4e-180
GCACAAAAAGAAGACTCAAGGCTGAGAATAGAGCTGACGTCTTGTGACCCAGACCCAGCCCTAAACACAAGGTGGCACTAAAGGGGTTG

GAAGTCTGTGGTGTACCGGGGTATCCACAGTCACAACAAAACTGCAATCCACGCAGAGTCCTAACTATTTCAGCTAACTCCCTGCCCAC

ACACTAAAGGCCTAGCAAAAGGCCCATTTCTGGGCATAAAAC
```
